# Molecular Evolution and Adaptation of Livestock-Associated Methicillin-Resistant Staphylococcus aureus (LA-MRSA) Sequence Type 9

**DOI:** 10.1128/mSystems.00492-21

**Published:** 2021-06-22

**Authors:** Fangyou Yu, Astrid V. Cienfuegos-Gallet, Marcus H. Cunningham, Ye Jin, Bingjie Wang, Barry N. Kreiswirth, Liang Chen

**Affiliations:** a Department of Clinical Laboratory, Shanghai Pulmonary Hospital, Tongji University School of Medicine, Shanghai, China; b Shanghai Key Laboratory of Tuberculosis, Shanghai Pulmonary Hospital, Tongji University School of Medicine, Shanghai, China; c First Affiliated Hospital, Zhejiang University School of Medicine, Hangzhou, Zhejiang, China; d Center for Discovery and Innovation, Hackensack Meridian Health, Nutley, New Jersey, USA; e Department of Medical Sciences, Hackensack Meridian School of Medicine, Nutley, New Jersey, USA; University of Southampton

**Keywords:** ST9, evolution, genomic analysis, antimicrobial resistance, virulence factors, LA-MRSA, pathogenicity island

## Abstract

Livestock-associated methicillin-resistant Staphylococcus aureus (LA-MRSA) sequence type 9 (ST9) has emerged and disseminated in Asia. It is associated with colonization or infection in both humans and animal hosts; however, the genetic factors underpinning its adaptation to animal and human population remain to be determined. Here, we conducted a genomic analysis of 191 ST9 S. aureus genomes collected from 12 different countries, including 174 genomes retrieved from public databases and 17 sequenced in this study. *In silico spa* typing, staphylococcal cassette chromosome *mec* (SCC*mec*) typing, and antimicrobial resistance and virulence gene mining were conducted, and the temporal phylogenetic signal was assessed by Bayesian inference. Our results point toward a human methicillin-susceptible S. aureus (MSSA) origin of ST9 that evolved approximately 2 centuries ago. Three major genetic events occurred during ST9 host shift from human to animals: the loss of the immune evasion cluster genes (*scn*, *chp*, and *sak*), which were reported to contribute to virulence in human infections, the acquisition of the SaPIbov4-like element-encoding *vwb* gene, which is an animal-specific virulence factor responsible for the clotting of animal plasma, and the acquisition of antibiotic resistance genes, including SCC*mec*, quinolone resistance-determining region (QRDR) mutations, and a multidrug resistance genetic element (MDR_ST9_). Evidence of direct transmission of animal-adapted strains to human hosts also suggest that transmission could potentially reshape the resistance and virulence genetic pool in these isolates. The rapid clonal expansion of MDR ST9 strains in mainland China and Taiwan highlights the increasing need for effective surveillance of antibiotic consumption in animal husbandry to control antimicrobial resistance spread.

**IMPORTANCE** Staphylococcus aureus sequence type 9 (ST9) is the main LA-MRSA clone spreading in the Asian continent. It can colonize and cause mild to severe infections both in animal and humans. Previous work described its genotypic characteristics; however, the molecular history of global spread of ST9 strains remains largely unclear. We conducted a detailed analysis of genomic evolution of global ST9 strains and identified key genetic changes associated with its adaptation to specific hosts. Our results suggest that the ST9 clone originated from human-adapted strains, which lost genes related to the evasion of the immune system. The introduction of ST9 strains in animal populations was aligned with the acquisition of animal-specific virulent factors and mobile elements harboring multiple antimicrobial resistance genes, especially in isolates from mainland China and Taiwan.

## INTRODUCTION

Methicillin-resistant Staphylococcus aureus (MRSA) is a common nosocomial and community-associated pathogen that can colonize or cause infections in both human and animals. Livestock-associated MRSA (LA-MRSA) has recently emerged and disseminated in Europe and North America. Since the first report of a human case of LA-MRSA in a 6-month-old infant in a pig farming family in the Netherlands in 2005 (1), an increasing number of cases of LA-MRSA have been described globally. Molecular screening among people with close contact with pigs and calves before hospital admission in areas with a high density of pig farming has shown 78% LA-MRSA carriage among MRSA-positive patients (2). Currently, clonal complex 398 (CC398) is the most predominate LA-MRSA clone in North America and Europe. Recent studies have identified CC398 LA-MRSA in 24% of MRSA isolates from Netherlands (3) and more than 10% in Belgium, Denmark, Spain and Slovenia (4).

In Asia, in contrast, sequence type 9 (ST9) is the most frequent LA-MRSA detected in pig farms and food products of animal origin, including mainland China (5, 6), Taiwan (7), and Malaysia (8). The ST9 strains are typically multidrug resistant and display virulence profiles different from those of other LA-MRSA clones (5, 9). ST9 LA-MRSA strains have also been found colonizing both animal and human hosts (10), and causing mild to severe infections in humans (11, 12). Several studies reported that ST9 constituted 70% to 95% of MRSA isolates detected in pigs and pig farm workers in China (7, 13–15). In addition, previous studies have identified highly similar genomic profiles between ST9 MRSA isolates from swine and humans, suggesting a cross-species (animal-to-human or human-to-animal) transmission of this clone (10, 11, 16, 17).

Interestingly, strains of this clone have been reported to cause infections in patients without a history of livestock contact (11, 12). The predominance of this clone in animals and its detection in human cases raise the questions of its origin and its ability to spread between animal and human hosts. The aim of this study was to unveil the genomic evolution of ST9 LA-MRSA and to probe the genetic characteristics associated with its adaptation to different hosts and its antimicrobial resistance and virulence profiles.

## RESULTS

### General characteristics of ST9 strains.

To probe the molecular evolution of ST9 strains, we analyzed publicly available genomes at the time of the study (January 2021) (*n* = 174) and 17 additional genomes from our collection, including ST9 strains collected globally between 1941 and 2019 from human and animal sources. These ST9 strains were isolated from swine (*n* = 110), human (*n* = 34), bovine (*n* = 12), meat (*n* = 11), other animal (*n* = 10), and unknown (*n* = 13) sources. The isolates were mostly collected in mainland China (*n* = 140), followed by Taiwan (*n* = 8), Ghana (*n* = 7), Germany (*n* = 6), the United States (*n* = 6), Czech Republic (*n* = 2), and Argentina, Colombia, Australia, Switzerland, and the United Kingdom (one isolate each). The 140 isolates from mainland China were collected from 13 of 31 different provinces, autonomous regions, or municipalities, with wide geographic distributions (see Fig. S1 in the supplemental material).

10.1128/mSystems.00492-21.1 FIG S1 Isolate map depicting the collection sites of ST9 isolates around the world. Yellow circles denote the 13 provinces, autonomous regions, or municipalities in China where the ST9 isolates were collected. Pie graphs show host distribution in selected countries (≥6 isolates), while the donut graphs demonstrate the distribution of MSSA or MRSA (SCC*mec* type) within each host. Download FIG S1, EPS file, 2.0 MB. Copyright © 2021 Yu et al. 2021 Yu et al. https://creativecommons.org/licenses/by/4.0/ This content is distributed under the terms of the Creative Commons Attribution 4.0 International license.

The 191 isolates belonged to 22 *spa* types, with *spa* t899 being the most frequent type (151/191, 79.1%). Eighty-one percent (122/151) *spa* t899 strains were identified in isolates from animal, while 16.5% (25/151) were from humans. Ninety-one percent (137/151) of *spa* t899 isolates were from mainland China or Taiwan. Other *spa* types identified were 1430 (7/191), t29922 (2/191), t4132 (2/191), t099, t100, t1334, t193, t2700, t464, t526, t587, t800, t13493, t2315, t3446, and t4794 (one of each).

Seventy-nine percent (151/191) of ST9 genomes harbored *mecA*, and the most predominant staphylococcal cassette chromosome *mec* (SCC*mec*) type was XII or XII-like (135/151). These SCC*mec* XII-like variants either harbored additional *ccrA1/ccrB2* (*n* = 2) or lacked *mec* class C2 and *ccrC2* (*n* = 11) (Fig. S2). SCC*mec* XII or XII-like elements were detected in isolates from both animal (bovine, chicken, meat, and swine) and human sources. Eight isolates harbored SCC*mec* IV (four from meat samples), and five contained hybrid SCC*mec* IV+XII (all from meat samples). Two strains harbored SCC*mec* V (2/191) and were of human origin. Selected genetic characteristics of human and animal isolates are presented in Table 1.

**TABLE 1:** Characteristics of human and animal ST9 isolates

Characteristic	No. (%) of isolates		
	Animal (*n* = 143)	Human (*n* = 34)	Total (*n* = 191)
Region			
Africa (Ghana)	7 (4.9)	0 (0.0)	7 (3.7)
America (Argentina, Colombia, USA)	4 (2.8)	4 (11.8)	8 (4.2)
Asia (China and Taiwan)	120 (83.9)	28 (82.35)	148 (77.5)
Europe (Czech Republic, Denmark, Germany, Poland, Switzerland)	12 (8.39)	1 (2.94)	13 (6.8)
Australia	0 (0.0)	0 (0.0)	1 (0.5)
Unknown	0 (0.0)	1 (2.9)	14 (7.3)^a^
SCC*mec*			
IV+XII	5 (3.5)	0 (0.0)	5 (2.6)
IV	4 (2.8)	0 (0.0)	4 (4.2)
V	0 (0.0)	2 (5.9)	2 (1.1)
XII and XII-like	115 (80.4)	19 (55.9)	135 (70.7)
Negative	18 (12.6)	13 (38.2)	40 (20.9)^a^
Major *spa* type			
t899	122 (85.3)	25 (73.5)	151 (79.1)
IEC (*scn-chp-sak*)	0.0 (0.0)	9 (26.47)	15 (7.9)^a^
SaPIbov4-like element	133 (93.0)	25 (73.5)	165 (86.4)
MDR_ST9_	120 (83.9)	26 (76.5)	147 (76.9)
QRDR mutation			
*parC*_S80F	137 (95.8)	27 (79.41)	170 (89.0)
*gyrA*_S84A	112 (78.3)	20 (58.8)	133 (69.6)

10.1128/mSystems.00492-21.2 FIG S2 Structure of staphylococcal chromosome *mec* (SCC*mec*) type XII and XII-like elements. Eleven of 13 XII-like SCC*mec* lacked ∼20 kb compared with XII SCC*mec*, including the region encoding *ccrC2.* Light blue shading represents regions of homology, and green shading denotes inversely displayed regions of homology. Orange arrows, hypothetical proteins; yellow arrows, transposases; red arrows, resistance genes; dark blue arrows, chromosome cassette recombinases; light pink arrows, other functions. Download FIG S2, EPS file, 0.5 MB. Copyright © 2021 Yu et al. 2021 Yu et al. https://creativecommons.org/licenses/by/4.0/ This content is distributed under the terms of the Creative Commons Attribution 4.0 International license.

### Virulence factor-encoding genes in ST9 genomes.

We also investigated the presence of several virulence factor genes, including those for capsule, adhesins, secreted enzymes, toxins, and immune evasion, to analyze the presence of particular virulence profile in ST9 strains. All ST9 genomes harbored 12 genes of the capsular serotype 8 (*cap8*) cluster, the adhesin gene *ebp*, and the polysaccharide intercellular adhesion locus genes *icaA*, *icaB*, *icaC*, *icaD*, and *icaR* (except that two were negative for *icaB* and *icaC*) (Data Set S1). Only a subset of isolates harbored the adhesion genes *clfA* (71.7%; *n* = 137) and *clfB* (58.1%; *n* = 111), most of which were from animal sources (109/137 and 91/111, respectively). Genes encoding secreted enzymes were identified in nearly all isolates, including those for aureolysin (*aur*) (100%), serine proteases (*sspA*, *sspB*, and *sspC*) (100%), lipase (*geh*) (99.5%; *n* = 190), and hyaluronidase (*hys*) (95.3%; *n* = 182).

10.1128/mSystems.00492-21.5 DATA SET S1 Accession numbers, general characteristics, antimicrobial resistant genes, and virulence factors in the ST9 genomes. Download Data Set S1, XLSX file, 0.1 MB. Copyright © 2021 Yu et al. 2021 Yu et al. https://creativecommons.org/licenses/by/4.0/ This content is distributed under the terms of the Creative Commons Attribution 4.0 International license.

Most ST9 genomes carried hemolysin genes, including *hlb* (91.1%; *n* = 174), *hld* (99.5%; *n* = 190), *hlgA* (100%; *n* = 191), *hlgB* (100%; *n* = 191), *hlgC* (100%; *n* = 191), and *hly/hla* (99.5%; *n* = 190), which have been shown to play an important role in skin colonization and infection (18). ST9 genomes also encoded a pivotal virulence factor, the type 7 secretion system (T7SS), which has been found to contribute to membrane integrity and homeostasis in the presence of antimicrobial fatty acids (19). Identified genes of T7SS corresponded to four membrane proteins (*esaA*, *essA*, *essB*, and *essC*), one cytosolic protein (*esaB*), and two effector proteins (*esxA* and *esxB*). All ST9 genomes also harbored the iron uptake protein genes *isdA*, *isdB*, *isdC*, and *isdC*, while most isolates lacked the enterotoxin genes *sea*, *sec*, *selk*, *sell*, *selq* (0.5%; 1/191), and *seb* (2.6%, 5/186). Likewise, a gene encoding exfoliatin (*eta*) was found in only five isolates, including one from human and four from unknown sources. All isolates were negative for the Panton-Valentine leukocidin (PVL) genes *lukS-PV* and *lukF-PV*.

We also evaluated the presence of the immune evasion cluster (IEC) genes encoding staphylococcal complement inhibitor (*scn*), chemotaxis-inhibiting protein (*chp*), and staphylokinase (*sak*). These genes were found in ϕSa3 prophages and were reported to be a major mechanism of human-specific adaptation contributing to the increased virulence of ST398 (20, 21). Importantly, we found that the IEC genes were located in an ∼42-kb prophage similar to phage P282 sequence, which truncated the *hlb* gene, the common insertion site for ϕSa3 (Fig. S3). In total, 15 isolates were found to harbor IEC, nine of human origin and six of unknown origin.

10.1128/mSystems.00492-21.3 FIG S3 Genetic structure of ϕSa3 harboring the immune evasion cluster genes (*scn*, *chp* and *sak*) in ST9 isolates. Genetic map of P282 is shown as a reference sequence. Light blue shading represents regions of homology, and green shading represents inversely displayed regions of homology. Pink arrows, IEC genes. Download FIG S3, EPS file, 0.7 MB. Copyright © 2021 Yu et al. 2021 Yu et al. https://creativecommons.org/licenses/by/4.0/ This content is distributed under the terms of the Creative Commons Attribution 4.0 International license.

### Staphylococcal type Vα genomic island in ST9 genomes.

**FIG 1:**
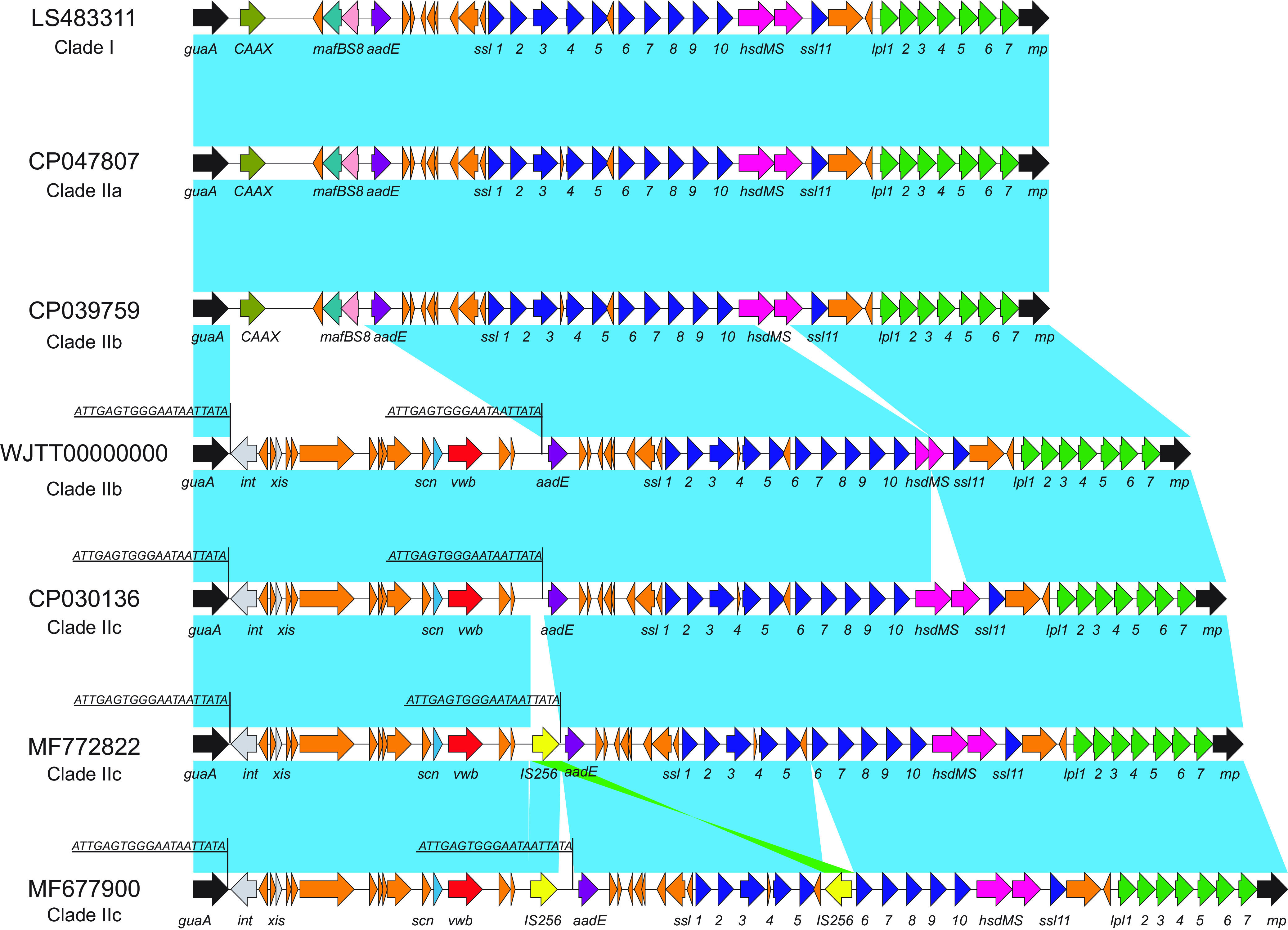
νSaα genomic island structures of isolates from clade I to IIc. Light blue shading represents regions of homology, while green shading denotes inverted displayed homologous regions. Orange arrows, hypothetical proteins; gray arrows, integrase and excisionase genes; yellow arrows, transposases; red arrow, *vwb* gene; light blue arrows, staphylococcal complement inhibitor; purple arrows, nucleotidyltransferase; dark blue arrows, staphylococcal superantigen-like protein genes; pink arrows, restriction-modification system; green arrows, lipoprotein-like genes. Direct repeats are underlined.

A previous study showed that ST9 strains harbor a νSaα genomic island, which carries a SaPIbov4-like element, in addition to the staphylococcal superantigen-like (*ssl1* to *ssl11*) and *lpl* tandem genes (22). This νSaα was designated as a type Vα genomic island based on the structure comparison with previously described type I to IV genomic islands and six additional novel νSaα genomic islands (type VI to XI) (22). The SAPIbov4-like element encodes a von Willebrand binding protein (*vwb*), an important animal-related virulence factor that can cause bovine and caprine plasma clotting (22). To investigate whether the type Vα genomic island was conserved in different ST9 strains, we analyzed its presence and sequence variation in the 191 genomes. We found that all isolates carry a νSaα structure, and 86.4% (165/191) of genomes contain the SaPIbov4-like element in νSaα. The sequence alignment showed that an ∼14-kb SAPIbov4-like element was inserted downstream of the glutamine synthase gene (*guaA*) and upstream of the aminoglycoside transferase gene (*aadE*) (Fig. 1). This SAPIbov4-like element had high frequencies (>90%) in swine (102/110), bovine (11/12), chicken (2/2), livestock farm (7/7), and meat (11/11) samples but a lower frequency (25/34; 73.5%) in human samples.

### Antimicrobial resistance genes in ST9 genomes.

To understand how the ST9 evolution could be driven by antibiotic selection pressure, we investigated the presence and colocalization of antibiotic resistance genes in ST9 genomes. Most ST9 isolates harbor multiple antimicrobial resistance genes, encoding resistance to β-lactams, aminoglycosides, lincosamides, chloramphenicol, tetracyclines, and quinolones. β-Lactam resistance was mainly mediated by *mecA* (79.1%; *n* = 151), and 83.8% of isolates also harbored a penicillin-hydrolyzing class A β-lactamase (*blaZ*) (83.8%; *n* = 160). Other common resistance genes included *lnu*(B) (75.4%; *n* = 144; encoding resistance to lincosamide), *lsa*(E) (75.9%; *n* = 145; lincosamide/streptogramin resistance) and *erm*(C) (60.7%; *n* = 116; macrolide resistance). Additional genes found in CC398 LA-MRSA isolates were also frequently found in the ST9 genomes, such as *fexA* (encoding a chloramphenicol/florfenicol efflux major facilitator superfamily [MFS] transporter) (69.1%; *n* = 132) (23). Most ST9 isolates carried the tetracycline resistance gene *tet*(L) (79.6%; *n* = 152), and one isolate (1/191) carried *tet*(M), which is also found in CC398 isolates (23). Seventy-six percent of ST9 isolates (*n* = 147) also harbored *dfrG*, a major determinant of trimethoprim resistance in human S. aureus infections (24). Only five isolates contained *dfrK*, an additional trimethoprim resistance gene.

Mutations associated with quinolone resistance in S. aureus were also commonly found in ST9 strains, with 89.0% (*n* = 170) presenting the double mutation *gyrA*_S84A/S84L/S84V and *parC*_S80F in the quinolone resistance-determining regions (QRDRs). A small number of isolates carried *rpoB* mutations, including H481N (4.71%; *n* = 9), which was reported to promote the emergence of stable rifampin-resistant small-colony variant (SCV) subpopulations with reduced susceptibility to vancomycin and daptomycin (25). Other rare *rpoB* mutations (I527M and S529L) were detected in two isolates.

**FIG 2:**
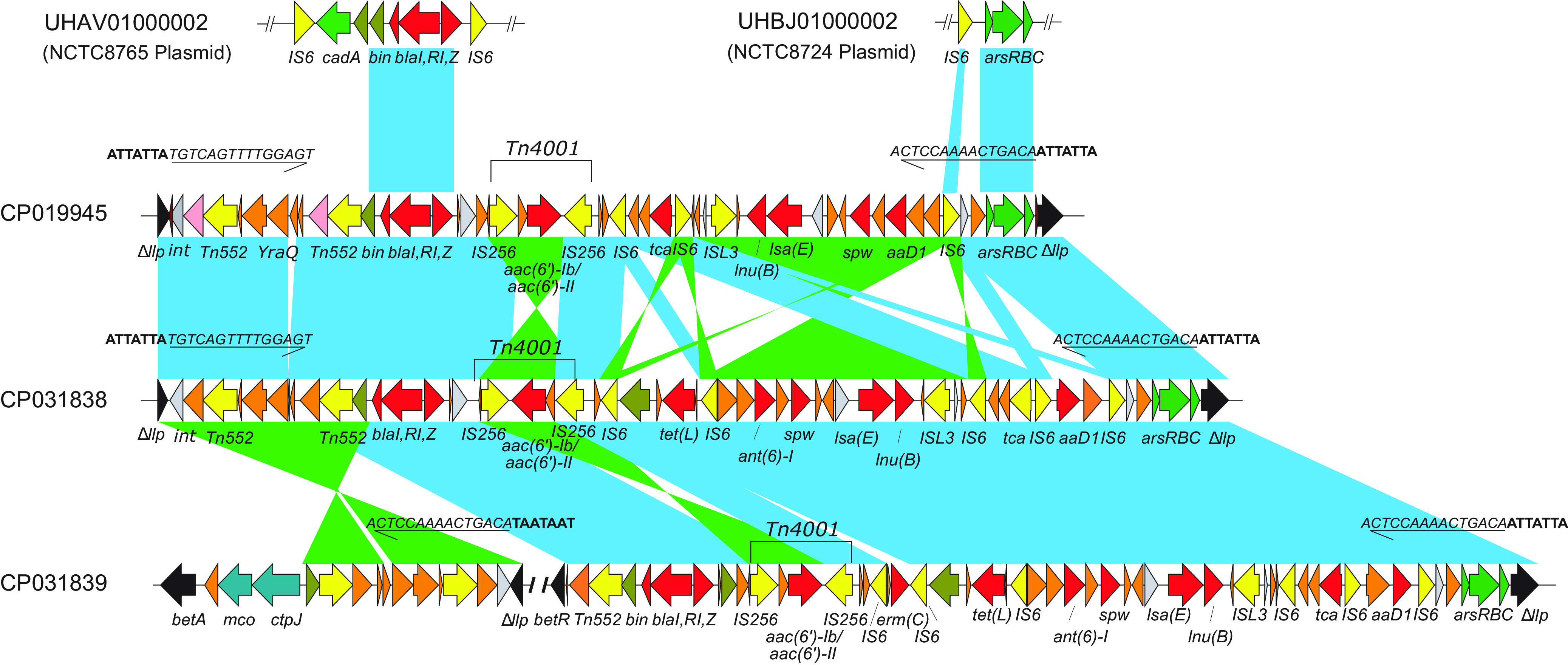
Structure of mobile genetic element MDR_ST9_, carrying the resistance genes *blaI*, *blaRI*, *blaZ*, *aa(6′)-Ie/aph(2′′)-Ia*, *tet*(L), *aaD1*, *spw*, *lsa*(E), and *lnu*(B). Light blue shading represents regions of homology; green shading denotes inversely displayed regions of homology. The MDR_ST9_ genetic element is inserted in *llp* (l-lactate permease) gene. Orange arrows, hypothetical proteins; gray arrows, integrase; yellow arrows, transposases; red arrows, resistance genes; olive arrows, recombinases; green arrows, arsenite detoxification; teal arrows, copper detoxification. The direct repeats are in bold while the invert repeats are in italics and underlined. The MDR_ST9_ in strain NX-T55 (CP031839) was separated into two regions separated by >210 kb, and the region between *int* and Tn*552* was inverted.

Further genome mining revealed that most antimicrobial resistance genes were chromosome borne. Besides the SCC*mec* region, the *fexA* transporter gene harboring Tn*558* was located downstream of a JAB domain-containing protein gene (locus tag D1G35_05855) on the chromosome. Interestingly, most of the resistance genes, including *aac(6*′*)-Ie/aph(2*′′*)-Ia*, *blaR1*, *blaZ*, *tet*(L), *lnu*(B), and *lsa*(E), were found to be colocalized within an ∼38- to 45-kb MDR region (tentatively named MDR_ST9_), inserted into a l-lactate permease gene (*llp*; locus tag E3T15_09260) with a 15-bp invert repeat (TGTCAGTTTTGGAGT) and a 7-bp target sequence duplicate (ATTATTA) on both termini (Fig. 2). This region was separated into two fragments in some genomes (e.g., strains S57 and NX-T55), presumably due to transposase mediated recombination. We also detected ST9 isolates harboring resistance plasmids (*blaI*, *blaRI*, *blaZ*, *arsR*, *arsB*, *arsC*, and *mco* genes), which could potentially be the origin of chromosome-borne resistant genes through recombination (Fig. 2).

The antimicrobial resistance profile of the 17 isolates available from our collection is presented in Data Set S2. These isolates were intermediate or resistant to methicillin (71.4%; *n* = 15), gentamicin (71.4%; *n* = 15), levofloxacin (76.2%; *n* = 16), moxifloxacin (76.2%; *n* = 16), erythromycin (52.4%; *n* = 11), and clindamycin (81.0%; *n* = 17). All 17 isolates were susceptible to ceftaroline, linezolid, daptomycin, vancomycin, and rifampin. The susceptibility testing results were in accordance with the results of the genome analysis, and most isolates were MDR (81.0%).

10.1128/mSystems.00492-21.6 DATA SET S2 Antimicrobial susceptibility results for 17 ST9 strains. Download Data Set S2, XLSX file, 0.01 MB. Copyright © 2021 Yu et al. 2021 Yu et al. https://creativecommons.org/licenses/by/4.0/ This content is distributed under the terms of the Creative Commons Attribution 4.0 International license.

### Phylogeographical context and comparative genomics of ST9 strains.

Next, we implemented a Bayesian phylogenetic inference to decipher the global evolutionary history of ST9 LA-MRSA and to identify key genetic changes associated to its adaptation to human and animal populations. Core genome analysis identified 6,955 core SNPs across 191 ST9 strains. The BactDating model of temporal phylogenetic signal showed convergence and was significantly better than the randomized dates model, with effective population sizes of greater than 200 (α, μ, and σ were >200). BactDating estimates that the most recent common ancestor (MRCA) of ST9 strains was around 1826 (95% confidence interval [CI], 1588 to 1912), approximately 200 years ago. The estimated mutation rate is 4.7 (95% CI, 3.6 to 5.9) single nucleotide polymorphisms (SNP)/genome/year, which is similar to the rates in other S. aureus lineages (26).

**FIG 3:**
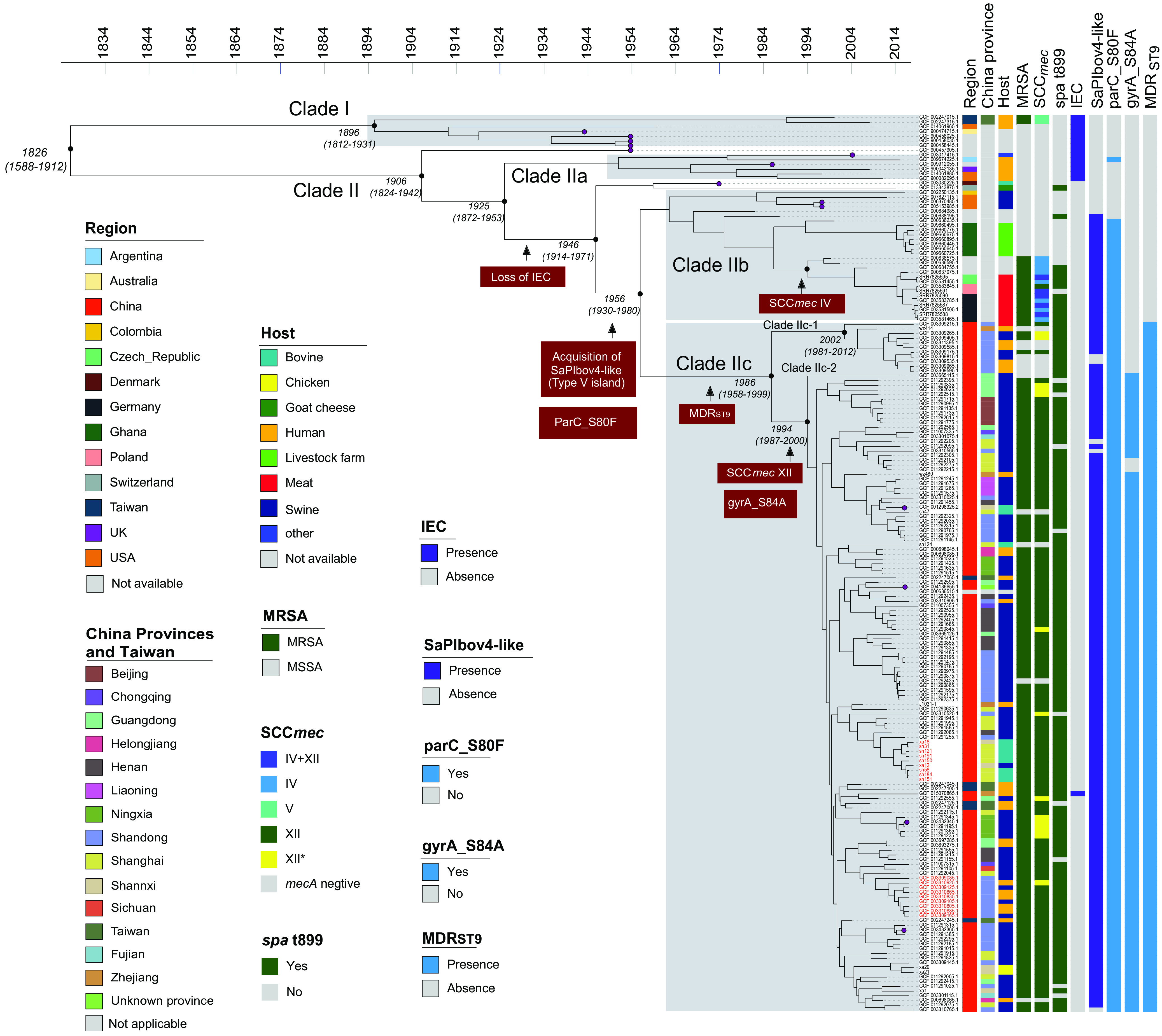
Phylogenetic analysis of 191 genomes of ST9 strains. Colors in columns illustrated region of origin, mainland China province and Taiwan, host, presence of *mecA*, SCC*mec*, *spa* type, the immune evasion cluster (EIC) genes, the SaPIbov4-like element carrying the *vwb* and *scn* genes, QRDR mutations (*parC* and *gyrA*), and the MDR_ST9_ element. Relevant evolutionary events are displayed in red boxes, and selected divergence time and 95% CIs are shown at the nodes. Purple tips indicate completely closed genomes. Strain names in red font indicate clusters of strains with possible interhost transmissions (<20 SNP).

Our analysis divided the 191 genomes into two main clades (I and II) (Fig. 3). The ancestral clade I (*n* = 7) originated around 1894 and included isolates from human (3/7) and unknown (4/7) sources collected in Taiwan (*n* = 2), Australia (*n* = 1), and the United States (*n* = 1). Most isolates (5/7) were MSSA and belonged to diverse *spa* types: t2700, t4358, and t4522. Two isolates harbored SCC*mec* type V.

The clade II strains can be divided into three subclades (IIa, IIb, and IIc) and a few singletons (*n* ≤ 2), with cluster IIc containing the largest number of genomes (*n* = 147) from mainland China and Taiwan. Clade IIa comprised only MSSA isolates, which were of human origin (5/6), collected in several countries around the world, including Argentina, the United States, and the United Kingdom. The *spa* types identified were t099, t100, t193, t464, and t587. Interestingly, most clade I and IIa (93.3%; 14/15) strains harbored the aforementioned IEC genes, *scn*, *chp*, and *sak*. The fact that we detected human MSSA isolates harboring the human complement evasion-associated IEC genes in the ancestral clades suggests a possible MSSA human origin of ST9 LA-MRSA clones and the change of human-specific virulence factors during the host shift to animals.

The separation of clade IIb/IIc from clade IIa correlated with the loss of the IEC genes (*scn*, *chp*, and *sak*), followed by the acquisition of the SaPIbo4-like element in the backbone of νSaα genomic island (type V) (Fig. 1) and the acquisition of QRDR mutations (*parC*_S80F) in ∼1956 (Fig. 3).

Clade IIb (*n* = 28) included isolates from animal sources, i.e., livestock farms (*n* = 7), meat (*n* = 10), swine (*n* = 4), and unknown origin (*n* = 7), collected in Europe (Czech Republic, Germany, and Poland), Africa (Ghana), and the Americas (United States and Colombia), and included both MSSA (14/28) and MRSA (14/28) isolates. The SaPIbov4-like element was present in 23/28 isolates, which were mostly from animal (17/23) sources. SCC*mec* was later acquired in the background of the SaPIbov4-like isolates. MRSA isolates (14/28) carried SCC*mec* IV (8/14), IV+XII (5/14), and XII (1/14), belonged to *spa* type t899 (11/14), and were mainly from European countries (10/14). MSSA isolates within this clade (14/28) belonged to seven different *spa* types, with t1430 (7/14) being the most common. These isolates were collected from Asia, Ghana (*n* = 7), the United States (*n* = 3), and Colombia (*n* = 1).

The largest subclade, clade IIc, emerged in ∼1986 and contained 147 genomes, exclusively from mainland China and Taiwan. The estimate of divergence time is consistent with the result (around 1987) of a recent ST9 genomic study in China (16). The isolates were mostly MDR strains, carrying aminoglycoside, β-lactam, macrolide, lincosamide, streptogramin, quinolone, chloramphenicol, tetracycline, and trimethoprim resistance genes (Fig. S4). In addition, genomes from clade IIc showed a highly similar resistance profile, and all carried the above-described MDR_ST9_ genetic element(s), supporting the hypothesis of clonal expansion. Almost all isolates from clade IIc carried an SaPIbov4-like element (98.7%; 145/147) and were recovered from both animal (118/145) and human (26/145) sources.

10.1128/mSystems.00492-21.4 FIG S4 Virulence factors and antimicrobial resistance genes detected in ST9 genomes. Only genes found in >10 isolates are presented. Download FIG S4, TIF file, 0.6 MB. Copyright © 2021 Yu et al. 2021 Yu et al. https://creativecommons.org/licenses/by/4.0/ This content is distributed under the terms of the Creative Commons Attribution 4.0 International license.

This clade, clade IIc, further diverged in two subclades, clades IIc-1 and IIc-2. The clade IIc-1 (*n* = 11) strains originated around 2002; all belonged to *spa* t899 (11/11) and were from both human (6/11) and swine (4/11) sources, including both MSSA (7/11) and MRSA (4/11, all SCC*mec* XII) isolates. Clade IIc-2 is the major sublineage currently disseminating in mainland China. This subclade originated around 1994 and rapidly disseminated into multiple provinces in China. No apparent introducing time could be determined for individual provinces. Genomes of isolates from different provinces frequently clustered together, despite the presence of some small clusters of closely related strains originating from the same provinces. The results suggested extensive ST9 isolate exchange in different regions in China, presumably due to frequent animal trade, as suggested in a previous study (16). Clade IIc-2 includes isolates from swine (*n* = 102), bovine (*n* = 11), chicken (*n* = 2) and human (*n* = 20) sources. Most clade IIc-2 strains contained a unique QRDR GyrA S84A mutation. Several genomes from animal and human sources were phylogenetically close and separated by <20 core SNPs and contained the same resistance and virulence genes, suggesting the likelihood of animal-to-human (or human-to-animal) transmissions (Fig. 3). Clade IIc-2 is primarily composed of MRSA isolates (131/136), belonging to *spa* t899 (122/131). However, in contrast to the SCC*mec* IV-*spa* t899 European isolates in clade IIb, isolates from clade IIc were SCC*mec* XII-*spa* t899 (121/122). These results suggested that the *spa* t899 strains in clades IIb and IIc each independently acquired SCC*mec* IV and XII. The presence of SaPIbov4-like is also common within this subclade (133/136), and the isolates with the broad MDR profile are found exclusively in this clade (Fig. S4).

## DISCUSSION

We detected three major genetic events along the evolutionary history of ST9: the loss of the IEC genes (*scn*, *chp*, and *sak*), which were reported to contribute to virulence in human infections, the acquisition of the SaPIbov4-like element-encoding *vwb* gene, which is an animal-specific virulence factor responsible for the clotting of animal plasma, and the acquisition of antibiotic resistance genes, including SCC*mec*, QRDR mutations and the MDR_ST9_ genetic elements.

First, all human MSSA isolates from the ancestral clade carried a ϕSa3 β-hemolysin-converting prophage, harboring the genes implicated in immune evasion (*scn*, *chp*, and *sak*). In contrast, isolates from clade IIb/IIc carried an intact β-hemolysin gene and were negative for ϕSa3, supporting the role of *scn*, *chp*, and *sak* as specific mechanisms of human adaptation. These three genes have a major role in complement evasion: the staphylococcal complement inhibitor (*scn*) binds to C3 convertases, preventing the activation of all three complement pathways (27); the chemotaxis inhibitory protein (*chp*) binds to C5aR1 and FPR1, thereby blocking the recognition of C5a and fMLF chemoattractants (28); and the staphylokinase (*sak*) activates plasminogen into plasmin, which is a serine protease bound to staphylococcal membrane which disrupts opsonization and phagocytosis through degradation of C3b and IgG, and it also blocks the cytolytic effect of human α-defensins (29).

Notably, previous studies showed that these virulence factors were specific for human hosts (30). Among them, SCN inhibits the alternative pathways exclusively found in humans (27), while the human chemotaxis-inhibiting protein had much lower capacity for binding to animal neutrophils, with 30-fold-reduced activation in mouse compared to human neutrophils (28). Similarly, bacterial plasminogen activators (PA) (such as streptokinase or staphylokinase) have a restricted ability to cleave different animal plasminogens (31). In addition, previous studies showed that the β-hemolysin-converting prophages were almost exclusively found in human isolates but absent from animal isolates (32). The results are consistent with previous studies showing that the IEC-harboring ϕSa3 β-hemolysin prophages were closely associated with S. aureus strains from humans (33, 34). Our results reiterate the loss of IEC is a major molecular event underlying the host shift from humans to animals during the molecular evolution of ST9 strains.

The second major event in the evolution of ST9 was the acquisition of the SaPIbov4-like element in the backbone of the νSaα genomic island (type V). This SaPIbov4-like element harbored an animal-specific virulence factor gene, the *vwb* gene, which encodes a homolog of the von Willebrand factor binding protein (35). Several *vwb* alleles showed species-specific coagulation activities in animals through unique N-terminal motifs which activate bovine and equine prothrombin, as an important animal host adaptation mechanism (36). However, the effect of SaPIbov4-encoding *vwb* on plasma clotting has been evaluated only in bovine and caprine plasma (22, 36). Its function in porcine plasma has not been fully studied, and future work is needed to understand its role in swine pathogenesis. Our analysis showed the acquisition of *vwb* in isolates from clade IIb and IIc, which contained mainly animal isolates. The SaPIbov4-like element was acquired by several *spa* types, initially in MSSA *spa* t1430 and later in MRSA *spa* t899 from clade IIb and clade IIc. This element also carried a second *scn* variant, which has 52.1% similarity to *scn* (SCIN-A) encoded in ϕSa3. A previous study reported the identification of an equine *scn* (eqSCIN) variant in prophage ϕSaeq1, detected in different lineages of S. aureus exclusively isolated from horses (37). Remarkably, this variant inhibits C3 convertases from horses but also is a potent inhibitor of human and pig complement (37). However, the role of this *scn* in interfering complement function from different human and animal hosts remains unclear and deserves future studies.

The third notable event was the acquisition of multiple resistance genes, including SCC*mec*, QRDR mutations, and the MDR_ST9_ element(s). Interestingly, the acquisition of SCC*mec* and QRDR mutations correlated with the emergence of *spa* t899 in ST9 strains. The phylogenomic analysis revealed an ancestral clade composed of MSSA human isolates of diverse *spa* types and a most recent clade composed of MRSA animal isolates of predominantly *spa* t899. In addition to our analysis, previous molecular typing reports of MRSA isolates collected from pigs and pig industry-related workers in China have also showed the predominance of ST9 and ST9 single-locus variant (SLV) *spa* t899 strains (6, 10, 13, 14). In contrast to our findings of *spa* t899-SCC*mec* XII predominance among ST9, isolates from previous work were *spa* t899-SCC*mec* III and SCC*mec* IV. These findings also support a human MSSA origin of ST9 MRSA, with independent acquisition of SCC*mec* elements in the background of different *spa* types.

Multidrug resistance was also a hallmark of animal strains. Human isolates in clade I and IIa contained only the resistance genes *lmrS*, *mepA*, *fosB*, *tet38*, *blaZ*, and *cadD*, and the clade IIb isolates (predominantly of animal origin) had obtained multiple antimicrobial resistance genes, including *mecA*, *vgaA*, *qacG*, QRDR mutations (*parC*_S80F), *tetK*, and *str*. The clade IIc isolates, including isolates from human and animal sources, carried the largest number of drug resistance genes, commonly those for resistance to methicillin (*mecA*), aminoglycosides [*aac(6′)-Ie*, *aadD1*, *ant(6′)-Ia*, *aph(2′′)-Ia*, *spw*], arsenite (*arsB* and *arsC*), copper (*mco*), fosfomycin (*fosB*), lincosamide (*lnuB* and *lsaE*), macrolides (*ermC*), chloramphenicol/florfenicol (*fexA*), quinolones (*gyrA*_S84A and *parC*_S80F), tetracycline (*tet38* and *tetL*), and trimethoprim (*dfrG*) and those for multidrug efflux MFS and MATE transporters (*lmrS* and *mepA*). However, resistance markers frequently associated with the LA-MRSA CC398 clone, such as the chromosomal gene *tet*(M), were nearly absent from the ST9 genomes, suggesting different antimicrobial resistance pressure in CC398 and ST9 strains.

Several of those genes, including *aa(6′)-Ie/aph(2′′)-Ia*, *blaR1*, *blaZ*, *tet*(L), *lnu*(B), and *lsa*(E), were located in the MDR_ST9_ chromosomal region(s). Our phylogenetic analysis showed that the acquisition of MDR_ST9_ region(s) was exclusively found in clade IIc from isolates from mainland China and Taiwan. A BLAST search of this element against NCBI database failed to detect similar sequences from other S. aureus clones, suggesting the MDR_ST9_ may originate through the molecular evolution of ST9 strains. Interestingly, the β-lactamase genes (*blaI*, *blaR*, and *blaZ*) and the arsenic resistant genes (*arsR*, *arsB*, and *arsC*) were found in plasmids from ancestor clade I strains (Fig. 2). Examination of MDR_ST9_ identified multiple genes with insertion elements (IS*256*, IS*6*, and IS*L3*) and a transposon (Tn*552*) (Fig. 2). We therefore hypothesized that MDR_ST9_ may have originated from the chromosomal integration of plasmid-borne genes (such as *blaI*, *blaR*, *blaZ*, *arsR*, *arsB*, and *arsC*) along with the acquisition of additional antimicrobial resistance genes encoding tetracycline (*tetL*) and aminoglycoside [*aac(6′)-Ib/aac(6′)-II*] resistance, through IS- or transposon-mediated transposition, as a result of evolution against the increased antibiotic selection pressures. This clone appeared to be widely disseminated in China, and genetically similar isolates were also reported recently in food surveillance for antimicrobial resistance from raw meat products in Hong Kong (38).

Moreover, we also detected evidence of interhost transmission of ST9 strains. Within clade IIc, at least one cluster of isolates from pig and human sources showed very close core SNPs (<20), in support of the likelihood of the transmission of the ST9 strains between human and animals (Fig. 3). These isolates were recently described in a pork production chain and were found to be carried by pigs and pig workers in several farms (40). Although we could not determine the direction of transmission, both animal-to-human and human-to-animal transmission could be possible (10, 11, 16, 17). Though not as common as animal-to-human transmission, human-to-animal transmission (i.e., reverse zoonosis) has been documented in S. aureus infections in livestock or companion animals (39). Interestingly, we detected one isolate carrying the IEC (*scn-chp-sak*) in this clade IIc. This strain was isolated from a patient with a bloodstream infection (BSI) without livestock contact and showed high *in vivo* and *in vitro* virulence, with virulence genetic profiles closely related to those of human-associated ST9 MSSA (12). Nonetheless, this isolate carried the SCC*mec* XII, MDR_ST9_, and the type V genomic island with the SaPIbov4-like element. Our results suggest that this isolate may have independently acquired human-specific factors (IEC) and caused severe infection in humans, molecular evidence of an MDR animal-adapted strain (identified by MDR_ST9_ and SaPIbov4-like elements) that could obtain hypervirulence through horizontal gene transfer. This finding warns of the potential emergence of multidrug-resistant and hypervirulent LA-MRSA strains.

Our results resembled the evolution of CC398, another successful LA-MRSA lineage, which originated in human as MSSA, spread to livestock, and later acquired methicillin resistance. In our study, the evolutionary analysis of ST9 genomes points to a human MSSA origin of ST9, which lost the IEC genes (*sak*, *chp*, and *scn*). The introduction of ST9 stains in animal populations was aligned with the acquisition of the SaPIbov4-like element, SCC*mec* (IV and XII), and multidrug resistance. The animal-adapted ST9 LA-MRSA strains showed the ability to infect humans, and the transmission from animal to human hosts could potentially reshape the resistance and virulence genetic pool in these isolates. The rapid clonal expansion of MDR ST9 in China and Taiwan highlights the increasing need for effective surveillance of antibiotic consumption in animal husbandry to control antimicrobial resistance spread.

## MATERIALS AND METHODS

### ST9 genomes.

Seventeen ST9 S. aureus isolates, collected in China from three provinces between 2011 and 2016, were included in this study. An additional 174 ST9 S. aureus genomes were retrieved from the NCBI whole-genome sequence database or short-read archive comprising publicly available genomes at the time of the study (January 2021). The accession number, host, isolation source, geographical origin, and genotype data are listed in Data Set S1.

### Antimicrobial susceptibility testing.

Antimicrobial susceptibility testing of the 17 ST9 isolates was evaluated using a Vitek-2 microbiology analyzer (bioMérieux, Marcy l’Etoile, France) in accordance with the manufacturer’s instructions. The MICs of 16 antimicrobial agents, including ampicillin, cefoxitin, ciprofloxacin, clindamycin, erythromycin, gentamicin, levofloxacin, linezolid, moxifloxacin, nitrofurantoin, oxacillin, rifampin, tetracycline, tigecycline, trimethoprim-sulfamethoxazole, and vancomycin, were determined, and the results were interpreted using Clinical and Laboratory Standards Institute guidelines (41). S. aureus strains ATCC 29213 and ATCC 25923 were used as quality controls.

### Whole-genome sequencing and analysis.

Whole-genome sequencing of the 17 ST9 isolates was carried out using the HiSeq 2500 sequencing platform (Illumina Inc., San Diego, CA), with 2 × 150 bp paired-end reads. The raw data were filtered using Trimmomatic v0.39 (42), followed by assembly using SPAdes v3.14 (43). Antimicrobial resistance genes were mined using AMRFinderPlus v3.9.8 (44). Virulence genes were identified by ABRicate v1.01 (https://github.com/tseemann/abricate) using the VFDB database (http://www.mgc.ac.cn/VFs/main.htm) with 95% identity and 90% query coverage cutoffs. SCC*mec* and *spa* types were determined by SCCmecFinder v1.2 (45) and spaTyper 1.0 (46), respectively. Comparative genomic analysis of mobile genetic structures was performed using Mauve v.2.4.0 (47, 48). In brief, the contigs from the genome assembly were ordered and oriented relative to a closed reference ST9 genome from each clade (Fig. 3) and then concatenated to form a pseudogenome with the Mauve contig mover (48). The resulting pseudochromosome was then aligned with the reference genome by progressive Mauve (49) to identify putative mobile genetic structures.

### Dating of ST9 strains.

Filtered reads from each isolate were mapped to the S. aureus ST9 reference genome (strain QD-CD9; accession number CP031838) by Snippy 4.4 (https://github.com/tseemann/snippy) using default settings. For genome assemblies downloaded from the NCBI WGS database, 10 million 150-bp paired-end reads were simulated using the wgsim (https://github.com/lh3/wgsim) algorithm from SAMtools (50) and were mapped to the reference genome using Snippy. Prophages were predicted using PHASTER (51), and repeated regions were examined using MUMmer (52). SNPs among prophages and repeated regions were excluded, as they reflect horizontal gene transfer events or are unable to be resolved by short-read sequencing. The recombination analysis was then performed using Gubbins v3.0.0 (53). The BactDating R package (54) was used to estimate node dates of ST9 strains. The recombination-corrected tree from Gubbins output (53) and the isolation time were used as the inputs in BactDating v1.1 (54), using a mixed model with 10^8^ iterations to ensure that the Markov chain Monte Carlo (MCMC) simulation was run for long enough to converge (the effective sample sizes of the inferred parameters α, μ, and σ were >200). Three BactDating replicates and one with a randomized tip date were conducted, and the convergence was evaluated with the Gelman diagnostic using the coda R package. The temporal signal significance was determined by comparing the first replicate model to the model with randomized tip date using the model compare function of the BactDating package (54). The resulting BactDating tree was then annotated using iTOL v5 (55).

### Data availability.

The raw reads of the 17 ST9 S. aureus genomes sequenced in this study were deposited in GenBank under BioProject accession no. PRJNA354234.

## References

[1] Voss A, Loeffen F, Bakker J, Klaassen C, Wulf M. 2005. Methicillin-resistant *Staphylococcus aureus* in pig farming. Emerg Infect Dis 11:1965–1966. doi:10.3201/eid1112.050428. 16485492 PMC3367632

[2] van de Sande-Bruinsma N, Leverstein van Hall MA, Janssen M, Nagtzaam N, Leenders S, de Greeff SC, Schneeberger PM. 2015. Impact of livestock-associated MRSA in a hospital setting. Antimicrob Resist Infect Control 4:2–7. doi:10.1186/s13756-015-0053-8. 25908965 PMC4407377

[3] de Greeff SC, Hoeing AF, Verduin CM. 2020. NethMap 2020: consumption of antimicrobial agents and antimicrobial resistance among medically important bacteria in the Netherlands in 2019/MARAN 2020: monitoring of antimicrobial resistance and antibiotic usage in animals in the Netherlands in 2019. National Institute for Public Health and the Environment, Bilthoven, the Netherlands.

[4] Kinross P, Petersen A, Skov R, Van Hauwermeiren E, Pantosti A, Laurent F, Voss A, Kluytmans J, Struelens MJ, Heuer O, Monnet DL, The European Human-LA-Mrsa Study Group. 2017. Livestock-associated meticillin-resistant S*taphylococcus aureus* (MRSA) among human MRSA isolates, European Union/European Economic Area countries, 2013. Euro Surveill 22:16-00696. doi:10.2807/1560-7917.ES.2017.22.44.16-00696. PMC5710135 29113628

[5] He W, Liu Y, Qi J, Chen H, Zhao C, Zhang F, Li H, Wang H. 2013. Food-animal related *Staphylococcus aureus* multidrug-resistant ST9 strains with toxin genes. Foodborne Pathog Dis 10:782–788. doi:10.1089/fpd.2012.1452. 23806146

[6] Ho PL, Chow KH, Lai EL, Law PYT, Chan PY, Ho AYM, Ng TK, Yam WC. 2012. Clonality and antimicrobial susceptibility of *Staphylococcus aureus* and methicillin-resistant *S. aureus* isolates from food animals and other animals. J Clin Microbiol 50:3735–3737. doi:10.1128/JCM.02053-12. 22895044 PMC3486263

[7] Fang H-W, Chiang P-H, Huang Y-C, HsinWei F, PoHsing C, YhuChering H. 2014. Livestock-associated methicillin-resistant *Staphylococcus aureus* ST9 in pigs and related personnel in Taiwan. PLoS One 9:e88826. doi:10.1371/journal.pone.0088826. 24551168 PMC3923820

[8] Neela V, Zafrul AM, Mariana NS, Van Belkum A, Liew YK, Rad EG. 2009. Prevalence of ST9 methicillin-resistant *Staphylococcus aureus* among pigs and pig handlers in Malaysia. J Clin Microbiol 47:4138–4140. doi:10.1128/JCM.01363-09. 19812280 PMC2786656

[9] Wang W, Liu F, Baloch Z, Zhang CS, Ma K, Peng ZX, Yan SF, Hu YJ, Gan X, Dong YP, Bai Y, Li FQ, Yan XM, Ma AG, Xu J. 2017. Genotypic characterization of methicillin-resistant Staphylococcus aureus isolated from pigs and retail foods in China. Biomed Environ Sci 30:570–580. doi:10.3967/bes2017.076. 28807097

[10] Bi Z, Sun C, Börjesson S, Chen B, Ji X, Berglund B, Wang M, Nilsson M, Yin H, Sun Q, Hulth A, Wang Y, Wu C, Bi Z, Nilsson LE. 2018. Identical genotypes of community-associated MRSA (ST59) and livestock-associated MRSA (ST9) in humans and pigs in rural China. Zoonoses Public Health 65:367–371. doi:10.1111/zph.12443. 29377579

[11] Chen C-J, Lauderdale T-LY, Lu C-T, Chuang Y-Y, Yang C-C, Wu T-S, Lee C-Y, Lu M-C, Ko W-C, Huang Y-C. 2018. Clinical and molecular features of MDR livestock-associated MRSA ST9 with staphylococcal cassette chromosome mecXII in humans. J Antimicrob Chemother 73:33–40. doi:10.1093/jac/dkx357. 29048488

[12] Jin Y, Yu X, Chen Y, Chen W, Shen P, Luo Q, Zhang S, Kong X, Zheng B, Xiao Y. 2020. Characterization of highly virulent community-associated methicillin-resistant *Staphylococcus aureus* ST9-SCC mec XII causing bloodstream infection in China. Emerg Microbes Infect 9:2526–2535. doi:10.1080/22221751.2020.1848354. 33174510 PMC7717876

[13] Ho J, O’Donoghue M, Guardabassi L, Moodley A, Boost M. 2012. Characterization of methicillin-resistant *Staphylococcus aureus* isolates from pig carcasses in Hong Kong. Zoonoses Public Health 59:416–423. doi:10.1111/j.1863-2378.2012.01473.x. 23057086

[14] Cui S, Li J, Hu C, Jin S, Li F, Guo Y, Ran L, Ma Y. 2009. Isolation and characterization of methicillin-resistant *Staphylococcus aureus* from swine and workers in China. J Antimicrob Chemother 64:680–683. doi:10.1093/jac/dkp275. 19684078

[15] Boost M, Ho J, Guardabassi L, O'Donoghue M. 2013. Colonization of butchers with livestock-associated methicillin-resistant *Staphylococcus aureus*. Zoonoses Public Health 60:572–576. doi:10.1111/zph.12034. 23279691

[16] Jiang N, Wyres KL, Li J, Fessler AT, Kruger H, Wang Y, Holt KE, Schwarz S, Wu C. 2021. Evolution and genomic insight into methicillin-resistant Staphylococcus aureus ST9 in China. J Antimicrob Chemother doi:10.1093/jac/dkab106. Epub ahead of print. 33822977

[17] Wan MT, Lauderdale TL, Chou CC. 2013. Characteristics and virulence factors of livestock associated ST9 methicillin-resistant Staphylococcus aureus with a novel recombinant staphylocoagulase type. Vet Microbiol 162:779–784. doi:10.1016/j.vetmic.2012.10.003. 23116588

[18] Katayama Y, Baba T, Sekine M, Fukuda M, Hiramatsu K. 2013. Beta-hemolysin promotes skin colonization by *Staphylococcus aureus*. J Bacteriol 195:1194–1203. doi:10.1128/JB.01786-12. 23292775 PMC3592002

[19] Kengmo Tchoupa A, Watkins KE, Jones RA, Kuroki A, Alam MT, Perrier S, Chen Y, Unnikrishnan M. 2020. The type VII secretion system protects *Staphylococcus aureus* against antimicrobial host fatty acids. Sci Rep 10:14838. doi:10.1038/s41598-020-71653-z. 32908165 PMC7481793

[20] Uhlemann A-C, Porcella SF, Trivedi S, Sullivan SB, Hafer C, Kennedy AD, Barbian KD, McCarthy AJ, Street C, Hirschberg DL, Lipkin WI, Lindsay JA, DeLeo FR, Lowy FD. 2012. Identification of a highly transmissible animal-independent *Staphylococcus aureus* ST398 clone with distinct genomic and cell adhesion properties. mBio 3:e00027-12. doi:10.1128/mBio.00027-12. 22375071 PMC3302565

[21] Kashif A, McClure JA, Lakhundi S, Pham M, Chen S, Conly JM, Zhang K. 2019. *Staphylococcus aureus* ST398 virulence is associated with factors carried on prophage ϕSa3. Front Microbiol 10:2219. doi:10.3389/fmicb.2019.02219. 31608039 PMC6771273

[22] Zhou W, Li X, Osmundson T, Shi L, Ren J, Yan H. 2018. WGS analysis of ST9-MRSA-XII isolates from live pigs in China provides insights into transmission among porcine, human and bovine hosts. J Antimicrob Chemother 73:2652–2661. doi:10.1093/jac/dky245. 29986036

[23] Kadlec K, Feßler AT, Hauschild T, Schwarz S. 2012. Novel and uncommon antimicrobial resistance genes in livestock-associated methicillin-resistant *Staphylococcus aureus*. Clin Microbiol Infect 18:745–755. doi:10.1111/j.1469-0691.2012.03842.x. 22509728

[24] Nurjadi D, Schäfer J, Friedrich-Jänicke B, Mueller A, Neumayr A, Calvo-Cano A, Goorhuis A, Molhoek N, Lagler H, Kantele A, Van Genderen PJJ, Gascon J, Grobusch MP, Caumes E, Hatz C, Fleck R, Mockenhaupt FP, Zanger P. 2015. Predominance of dfrG as determinant of trimethoprim resistance in imported *Staphylococcus aureus*. Clin Microbiol Infect 21:1095.E5–1095.E9. doi:10.1016/j.cmi.2015.08.021. 26344335

[25] Guérillot R, Gonçalves da Silva A, Monk I, Giulieri S, Tomita T, Alison E, Porter J, Pidot S, Gao W, Peleg AY, Seemann T, Stinear TP, Howden BP. 2018. Convergent evolution driven by rifampin exacerbates the global burden of drug-resistant Staphylococcus aureus. mSphere 3:e00550-17. doi:10.1128/mSphere.00550-17. 29404415 PMC5784246

[26] Uhlemann AC, McAdam PR, Sullivan SB, Knox JR, Khiabanian H, Rabadan R, Davies PR, Fitzgerald JR, Lowy FD. 2017. Evolutionary dynamics of pandemic methicillin-sensitive *Staphylococcus aureus* ST398 and its international spread via routes of human migration. mBio 8:e01375-16. doi:10.1128/mBio.01375-16. 28096484 PMC5241395

[27] Rooijakkers SHM, Ruyken M, Roos A, Daha MR, Presanis JS, Sim RB, van Wamel WJB, van Kessel KPM, van Strijp JAG. 2005. Immune evasion by a staphylococcal complement inhibitor that acts on C3 convertases. Nat Immunol 6:920–927. doi:10.1038/ni1235. 16086019

[28] De Haas CJC, Veldkamp KE, Peschel A, Weerkamp F, Van Wamel WJB, Heezius ECJM, Poppelier MJJG, Van Kessel KPM, Van Strijp JAG. 2004. Chemotaxis inhibitory protein of *Staphylococcus aureus*, a bacterial antiinflammatory agent. J Exp Med 199:687–695. doi:10.1084/jem.20031636. 14993252 PMC2213298

[29] Jin T, Bokarewa M, Foster T, Mitchell J, Higgins J, Tarkowski A. 2004. *Staphylococcus aureus* resists human defensins by production of staphylokinase, a novel bacterial evasion mechanism. J Immunol 172:1169–1176. doi:10.4049/jimmunol.172.2.1169. 14707093

[30] Sung JML, Lloyd DH, Lindsay JA. 2008. *Staphylococcus aureus* host specificity: comparative genomics of human versus animal isolates by multi-strain microarray. Microbiology (Reading) 154:1949–1959. doi:10.1099/mic.0.2007/015289-0. 18599823

[31] Gladysheva IP, Turner RB, Sazonova IY, Liu L, Reed GL. 2003. Coevolutionary patterns in plasminogen activation. Proc Natl Acad Sci U S A 100:9168–9172. doi:10.1073/pnas.1631716100. 12878727 PMC170890

[32] Resch G, François P, Morisset D, Stojanov M, Bonetti EJ, Schrenzel J, Sakwinska O, Moreillon P. 2013. Human-to-bovine jump of *Staphylococcus aureus* CC8 is associated with the loss of a β-hemolysin converting prophage and the acquisition of a new staphylococcal cassette chromosome. PLoS One 8:e58187. doi:10.1371/journal.pone.0058187. 23505465 PMC3594393

[33] Verkaik NJ, Benard M, Boelens HA, de Vogel CP, Nouwen JL, Verbrugh HA, Melles DC, van Belkum A, van Wamel WJ. 2011. Immune evasion cluster-positive bacteriophages are highly prevalent among human Staphylococcus aureus strains, but they are not essential in the first stages of nasal colonization. Clin Microbiol Infect 17:343–348. doi:10.1111/j.1469-0691.2010.03227.x. 20370801

[34] Cuny C, Abdelbary M, Layer F, Werner G, Witte W. 2015. Prevalence of the immune evasion gene cluster in Staphylococcus aureus CC398. Vet Microbiol 177:219–223. doi:10.1016/j.vetmic.2015.02.031. 25778546

[35] Novick RP. 2019. Pathogenicity islands and their role in staphylococcal biology. Microbiol Spectr 7:GPP3-0062-2019. doi:10.1128/microbiolspec.GPP3-0062-2019. PMC11257176 31172913

[36] Viana D, Blanco J, Tormo-Más MÁ, Selva L, Guinane CM, Baselga R, Corpa JM, Lasa Í, Novick RP, Fitzgerald JR, Penadés JR. 2010. Adaptation of *Staphylococcus aureus* to ruminant and equine hosts involves SaPI-carried variants of von Willebrand factor-binding protein. Mol Microbiol 77:1583–1594. doi:10.1111/j.1365-2958.2010.07312.x. 20860091

[37] de Jong NWM, Vrieling M, Garcia BL, Koop G, Brettmann M, Aerts PC, Ruyken M, van Strijp JAG, Holmes M, Harrison EM, Geisbrecht BV, Rooijakkers SHM. 2018. Identification of a staphylococcal complement inhibitor with broad host specificity in equid. J Biol Chem 293:4468–4477. doi:10.1074/jbc.RA117.000599. 29414776 PMC5868266

[38] Sapugahawatte DN, Li C, Yeoh YK, Dharmaratne P, Zhu C, Ip M. 2020. Swine methicillin-resistant *Staphylococcus aureus* carrying toxic-shock syndrome toxin gene in Hong Kong, China. Emerg Microbes Infect 9:1534–1536. doi:10.1080/22221751.2020.1785335. 32573344 PMC7473289

[39] Messenger AM, Barnes AN, Gray GC. 2014. Reverse zoonotic disease transmission (zooanthroponosis): a systematic review of seldom-documented human biological threats to animals. PLoS One 9:e89055. doi:10.1371/journal.pone.0089055. 24586500 PMC3938448

[40] Sun C, Chen B, Hulth A, Schwarz S, Ji X, Nilsson LE, Ma S, Sun Q, Bi Z, Wang Y, Bi Z, Wu C, Börjesson S. 2019. Genomic analysis of *Staphylococcus aureus* along a pork production chain and in the community, Shandong Province, China. Int J Antimicrob Agents 54:8–15. doi:10.1016/j.ijantimicag.2019.03.022. 30959181

[41] CLSI. 2020. Performance standards for antimicrobial susceptibility testing, 30th ed. CLSI supplement M100. CLSI, Wayne, PA.

[42] Bolger AM, Lohse M, Usadel B. 2014. Trimmomatic: a flexible trimmer for Illumina sequence data. Bioinformatics 30:2114–2120. doi:10.1093/bioinformatics/btu170. 24695404 PMC4103590

[43] Bankevich A, Nurk S, Antipov D, Gurevich AA, Dvorkin M, Kulikov AS, Lesin VM, Nikolenko SI, Pham S, Prjibelski AD, Pyshkin AV, Sirotkin AV, Vyahhi N, Tesler G, Alekseyev MA, Pevzner PA. 2012. SPAdes: a new genome assembly algorithm and its applications to single-cell sequencing. J Comput Biol 19:455–477. doi:10.1089/cmb.2012.0021. 22506599 PMC3342519

[44] Feldgarden M, Brover V, Haft DH, Prasad AB, Slotta DJ, Tolstoy I, Tyson GH, Zhao S, Hsu CH, McDermott PF, Tadesse DA, Morales C, Simmons M, Tillman G, Wasilenko J, Folster JP, Klimke W. 2019. Validating the AMRFINder tool and resistance gene database by using antimicrobial resistance genotype-phenotype correlations in a collection of isolates. Antimicrob Agents Chemother 63:e00483-19. doi:10.1128/AAC.00483-19. 31427293 PMC6811410

[45] Kaya H, Hasman H, Larsen J, Stegger M, Johannesen B, Allesøe L. 2018. SCCmecFinder, a web-based tool for typing of staphylococcal cassette chromosome mec in *Staphylococcus aureus* using whole-genome sequence data. mSphere 3:e00612-17. doi:10.1128/mSphere.00612-17. 29468193 PMC5812897

[46] Bartels MD, Petersen A, Worning P, Nielsen JB, Larner-Svensson H, Johansen HK, Andersen LP, Jarløv JO, Boye K, Larsen AR, Westh H. 2014. Comparing whole-genome sequencing with sanger sequencing for *spa* typing of methicillin-resistant *Staphylococcus aureus*. J Clin Microbiol 52:4305–4308. doi:10.1128/JCM.01979-14. 25297335 PMC4313303

[47] Darling ACE, Mau B, Blattner FR, Perna NT. 2004. Mauve: multiple alignment of conserved genomic sequence with rearrangements. Genome Res 14:1394–1403. doi:10.1101/gr.2289704. 15231754 PMC442156

[48] Rissman AI, Mau B, Biehl BS, Darling AE, Glasner JD, Perna NT. 2009. Reordering contigs of draft genomes using the Mauve aligner. Bioinformatics 25:2071–2073. doi:10.1093/bioinformatics/btp356. 19515959 PMC2723005

[49] Darling AE, Mau B, Perna NT. 2010. progressiveMauve: multiple genome alignment with gene gain, loss and rearrangement. PLoS One 5:e11147. doi:10.1371/journal.pone.0011147. 20593022 PMC2892488

[50] Li H, Handsaker B, Wysoker A, Fennell T, Ruan J, Homer N, Marth G, Abecasis G, Durbin R, 1000 Genome Project Data Processing Subgroup. 2009. The Sequence Alignment/Map format and SAMtools. Bioinformatics 25:2078–2079. doi:10.1093/bioinformatics/btp352. 19505943 PMC2723002

[51] Arndt D, Grant JR, Marcu A, Sajed T, Pon A, Liang Y, Wishart DS. 2016. PHASTER: a better, faster version of the PHAST phage search tool. Nucleic Acids Res 44:W16–W21. doi:10.1093/nar/gkw387. 27141966 PMC4987931

[52] Marçais G, Delcher AL, Phillippy AM, Coston R, Salzberg SL, Zimin A. 2018. MUMmer4: a fast and versatile genome alignment system. PLoS Comput Biol 14:e1005944. doi:10.1371/journal.pcbi.1005944. 29373581 PMC5802927

[53] Croucher NJ, Page AJ, Connor TR, Delaney AJ, Keane JA, Bentley SD, Parkhill J, Harris SR. 2015. Rapid phylogenetic analysis of large samples of recombinant bacterial whole genome sequences using Gubbins. Nucleic Acids Res 43:e15. doi:10.1093/nar/gku1196. 25414349 PMC4330336

[54] Didelot X, Croucher NJ, Bentley SD, Harris SR, Wilson DJ. 2018. Bayesian inference of ancestral dates on bacterial phylogenetic trees. Nucleic Acids Res 46:e134–e134. doi:10.1093/nar/gky783. 30184106 PMC6294524

[55] Letunic I, Bork P. 2019. Interactive Tree Of Life (iTOL) v4: Recent updates and new developments. Nucleic Acids Res 47:W256–W259. doi:10.1093/nar/gkz239. 30931475 PMC6602468

