# Supplementary figures and images for: Molecular Evolution and Adaptation of Livestock-Associated Methicillin-Resistant Staphylococcus aureus (LA-MRSA) Sequence Type 9
Source: mSystems. 2021 Jun 22;6(3):e00492-21. doi: 10.1128/mSystems.00492-21 (PMC8269235; doi:10.1128/mSystems.00492-21)

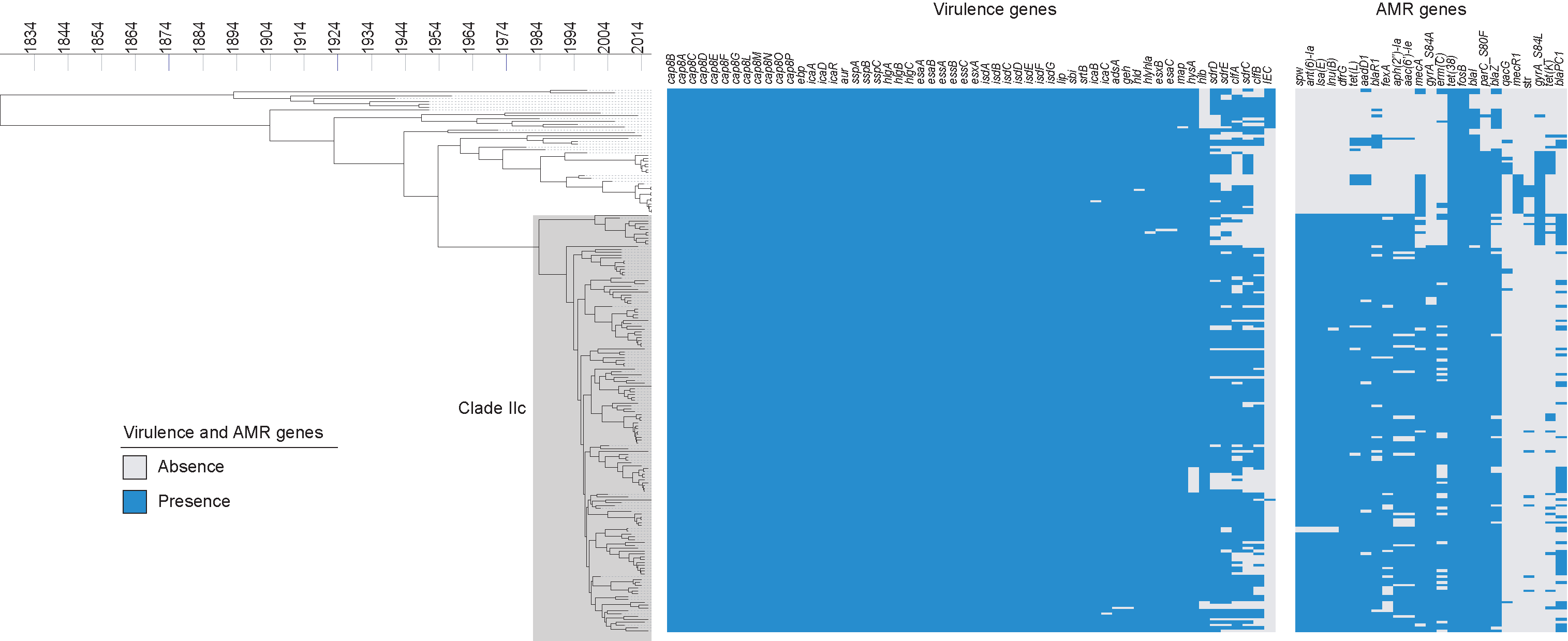

Supplement: FIG S4 [file msystems.00492-21-sf004.tif]
